# Central Pontine Myelinolysis With Carbamazepine-Induced Syndrome of Inappropriate Antidiuretic Hormone and Its Management: A Case Report and Literature Review

**DOI:** 10.7759/cureus.35816

**Published:** 2023-03-06

**Authors:** Abia Joseph, Tass Sayeed, Dev K Patel, Sanathan Aiyadurai, Zainab Shahbaz, Sambasiva Rao Mettela, Tulika Garg, Rishika Gadde, Datiobong Udoeyop, Aadil Khan

**Affiliations:** 1 Surgery, John F. Kennedy University School of Medicine, Illinois, USA; 2 Internal Medicine, Windsor University School of Medicine, Chicago, USA; 3 Internal Medicine, Smt. Nathiba Hargovandas Lakhmichand Municipal Medical College, Ahmedabad, IND; 4 Internal Medicine, Caribbean Medical University, Willemstad, CUW; 5 Internal Medicine, Katuri Medical College and Hospital, Tenali, IND; 6 Internal Medicine, Government Medical College & Hospital, Chandigarh, Chandigarh, IND; 7 Internal Medicine, Jawaharlal Nehru Medical College, Belgaum, IND; 8 Surgery, Richmond Gabriel University, Chicago, USA; 9 Internal Medicine, Lala Lajpat Rai Hospital, Kanpur, IND

**Keywords:** acute euvolemic hyponatremia, extrapontine myelinolysis (epm), bipolar affective disorder, antidiuretic hormone, osmotic demyelination syndrome (ods)

## Abstract

Aggressive treatment of hyper or hypoosmolar conditions can trigger osmotic demyelination syndrome. We describe the case of a 53-year-old male who began using carbamazepine to treat bipolar affective disorder and was later diagnosed with carbamazepine-induced syndrome of inappropriate antidiuretic hormone secretion. The patient’s mental state gradually improved once the hyponatremia was corrected using 3% normal saline and supportive therapy. The patient presented to the outpatient clinic with confusion and altered sensorium. Brain computed tomography showed diffuse cerebral atrophy and periventricular ischemia demyelination alterations, and magnetic resonance imaging showed an enhanced section in the brainstem that included the pons, suggesting osmotic demyelination alterations. Ventilatory support and supportive therapy were initiated, and hyponatremia was rectified. Although the patient did well with the treatment, his prognosis was still dismal, so he was sent home with instructions to follow up.

## Introduction

Osmotic demyelination syndromes, such as central pontine myelinolysis (CPM), cause damage to several brain regions, most frequently the pons. The fundamental pathophysiology is not hyponatremia per se but rather the quick correction (>12 mEq/L over 24 hours) of hyponatremia [1]. CPM can also occur in mild hyponatremia with slower correction rates. However, it usually happens when the correction of the blood sodium level surpasses 12 mEq/L/day [2]. It is essential to consider additional triggering variables, such as chronic liver disease, diabetes, and alcoholism. Although many presentations have been documented, patients often present with fast-progressing neurological symptoms and changes in mental status, dysphagia, and dysarthria [2-4]. This disease progresses quickly and is usually lethal. Imaging is used to confirm the diagnosis, and magnetic resonance imaging (MRI) is the recommended imaging modality as white matter hyperintensity, compared to computed tomography (CT), and hypoattenuation are visible on typical T2-weighted MRI [3,4]. Treatment of the underlying cause is combined with supportive care as management. More recent research has revealed a significantly improved prognosis, with a favorable outcome in roughly 33-50% of patients. In contrast, studies conducted before the mid-1980s showed a mortality rate of 90-100%. Around 24-39% of patients achieve resolution, and 16-34% of patients are independent in all activities of daily living [5-7]. In contrast, around 33-55% of patients will either require no care, need treatment ultimately, or pass away. Hyponatremia with concurrent hypokalemia, 114 mmol/L of severe hyponatremia, and a noticeable decline in alertness are indicators of poor results. Despite the delayed development of osmotic demyelination syndrome, the gradual reversal of hyponatremia, and the symptoms’ severity, CPM makes this case particularly noteworthy. Additionally, the difficulty of vigilance and follow-up is increased by the existence of a concurrent psychiatric disorder.

## Case presentation

A 53-year-old male was brought to the emergency department with chief complaints of increased urinary frequency, generalized weakness for one month, dizziness, and altered behavior for the past three days. His past medical history revealed chronic subdural hematoma for which he underwent decompressive craniectomy three years back and a history of bipolar affective disorder for the past 20 years. He was compliant with his medication which included carbamazepine 300 mg twice daily and lorazepam 2 mg once daily. On physical examination, blood pressure was 114/76 mmHg, pulse rate was 84/minute, and SpO_2_ was 97% on room air. He looked pale, icterus was present without pedal edema, and his Glasgow Coma Scale (GCS) score was 13/15. Systemic examination was within normal limits. He had no history of fever, dysuria, nocturia, or hematuria.

Routine abnormal biochemical parameters on admission are shown in Table 1. All investigations were suggestive of severe hyponatremia, and plasma osmolality was below 275 mOsm/kg. Complete urinalysis revealed high urine sodium (39 mEq/L) and urine osmolality (111 mOsm/kg). Endocrine and psychiatric opinions were obtained. After integrating clinical symptoms and lab investigation, a provisional diagnosis of drug-induced syndrome of inappropriate antidiuretic hormone (SIADH) was made. Immediately, carbamazepine was discontinued, and he was managed for severe hyponatremia over 24 hours using 3% normal saline at a rate of 0.5 mEq/L per hour and supportive therapy. He responded well to the treatment, and the electrolyte imbalance was successfully corrected, as shown in Table 1. He was discharged on the fifth day of hospital admission after improvement in his clinical symptoms. His psychiatric medication was also switched to tablet haloperidol 2.5 mg twice daily, melatonin 10 mg once a day, and quetiapine 25 mg as needed.

**Table 1:** Biochemical parameters during the course of hospitalization.

Parameter	Laboratory value on admission	Laboratory value on discharge
Serum sodium	104 (135–147) mmol/L	136 mmol/L
Serum potassium	4.29 (3.5–5.1) mmol/L	4.5 mmol/L
Serum calcium	4.24 (4.5–5.6) mg/dL	4.3 mg/dL
Urine sodium	323 (75–200) mmol/L	195 mmol/L
Urine potassium	34.9 (10–20) mmol/L	18 mmol/L
Urine calcium	3.72 (4.6–5.3) mg/dL	4.0 mg/dL

**Figure 1:**
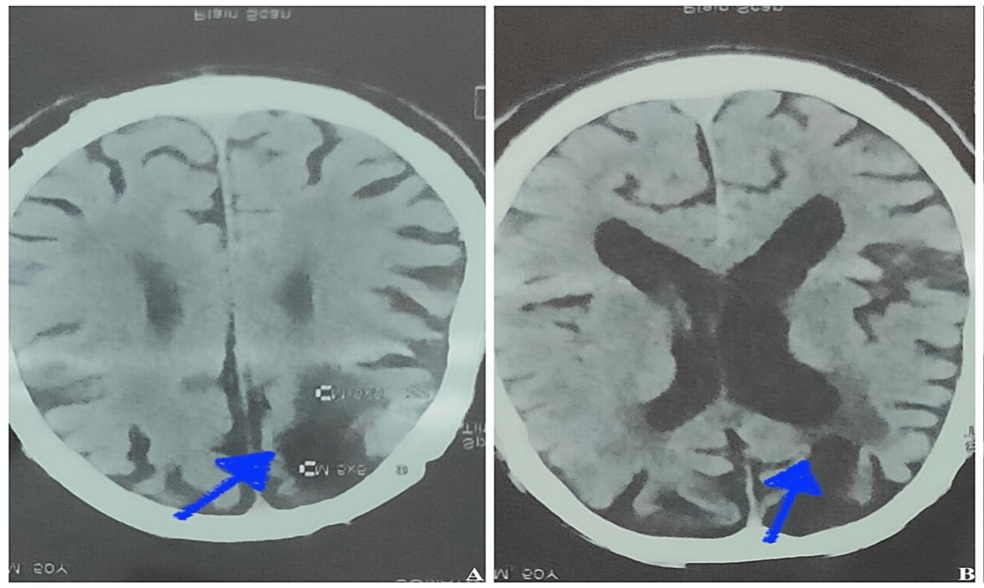
Gliosis in the right frontal and left parietal regions with periventricular ischemic demyelination (A, B).

**Figure 2:**
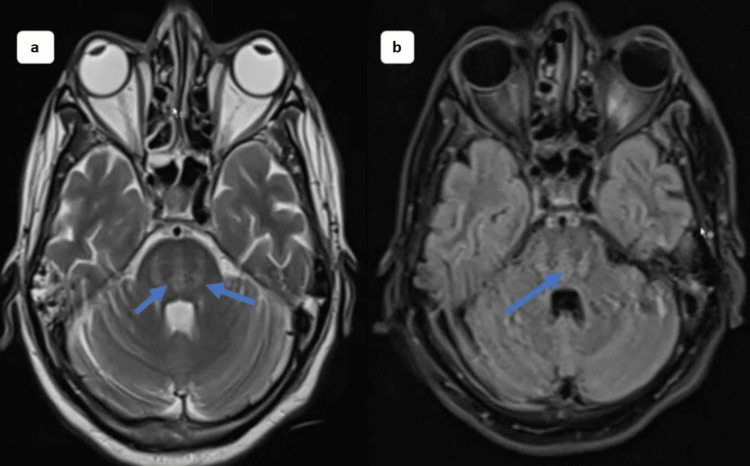
MRI brain demonstrating hyperintensity in the pons extending both sides of midline, suggesting demyelination (a, b). MRI: magnetic resonance imaging.

After two weeks of follow-up, he presented again with confusion and altered sensorium, followed by slurred speech and difficulty walking. Systemic examination revealed hyperreflexia, hypertonicity in all limbs, nystagmus, and ophthalmoplegia. He was admitted to the intensive care unit and intubated due to poor GCS and increased aspiration risk. He underwent a CT brain, which revealed gliosis in the right frontal and left parietal regions and diffuse cerebral atrophy with periventricular ischemic demyelination changes (Figures 1A, 1B). MRI revealed an enhancing lesion in the brainstem, including pons, suggestive of osmotic demyelination changes (Figure 2).

He received supportive management, and the electrolyte imbalance was corrected. A multidisciplinary approach was adopted in the care of the patient involving various specialties. He eventually responded to treatment and became conscious on day seven; however, rigidity and hyperreflexia persisted. He was discharged on continuous cycles of physical rehabilitation with regular follow-ups.

## Discussion

CPM is a clinically variable demyelinating disorder [8]. Extrapontine myelinolysis (EPM) or central and extrapontine myelinolysis are two terms used to describe the condition when de-myelination is observed outside of the pons [9]. The severity of the disease might range from incidental asymptomatic detection on imaging to coma or death. A typical combination of neuropsychiatric symptoms such as emotional lability, disinhibition, and other odd behaviors may be seen. The neurologic symptoms of CPM include generalized seizures, confusion, gait instability, and dysarthria [10]. The most frequent cause of CPM is a rapid correction of hyponatremia in patients with conditions that cause electrolyte stress, such as alcoholism, immunosuppression after transplantation, malnutrition with underlying illnesses, gastrointestinal disorders with acute electrolyte abnormalities, SIADH, renal disease, and high-intensity exercise [11,12]. Acute hyponatremia (48-hour development) typically results in a more severe patient presentation than chronic hyponatremia (more than 48-hour development). Still, chronic hyponatremia can indicate a higher risk for CPM because of compensatory mechanisms involving solute losses, which puts patients at higher risk for the development of CPM [13].

Water moves from the circulation into the brain during the acute phase of hyponatremia, causing brain cells to expand [14]. Brain cells rapidly lose inorganic osmolytes to restore normal cell volume in response to hyponatremia. The brain can swiftly store electrolytes to return to normal osmotic equilibrium if the hyponatremic status is rectified during this acute phase. Only the most vulnerable patients, such as menstruating women and patients with hypoxemia, will likely suffer from brain injury due to volume loss and deranged metabolites. If the hyponatremic state persists, brain cells must also evacuate organic osmolytes (such as taurine and glutamine) and electrolytes to fully return to normal cell volume. Compared to electrolytes, organic osmolytes move far more slowly from intracellular to extracellular volume [5].

For the brain to be in osmotic equilibrium with the blood, the intracellular osmolality of brain cells must be low in the chronic hyponatremic condition while brain cell volume is normal [9]. Hyponatremia must be corrected slowly to provide the brain cells enough time to reacquire both inorganic and organic osmolytes. Blood-brain barrier function may occasionally be impaired by osmotic stress brought on by the quick repair of chronic hyponatremia, which may indicate a loosening of vascular endothelial cells’ tight connections [6]. This procedure might give harmful chemicals (complement system) access to the myelin. It has also been suggested that vascular endothelial cells release other substances, such as plasminogen activators and cytokines, under osmotic stress that can harm myelin. Another explanation is that myelin degradation results from impaired potassium siphoning, which has recently been proposed for other demyelinating illnesses. This is supported by the discovery of structures resembling Rosenthal fibers within perivascular astrocytes [15].

Hyponatremia is a potential side effect of numerous neuropsychiatric drugs. About 10-25% of those with persistent mental illness may have primary polydipsia, which raises the risk of hyponatremia and early death [16]. Carbamazepine, serotonin reuptake inhibitors (SSRIs), tricyclic antidepressants, opioids, and polypharmacy with multiple antipsychotic drugs are the primary medication classes used daily in neuropsychiatry where hyponatremia is a side effect or reported to occur after use [17]. Other frequently prescribed drugs that raise the risk of hyponatremia include salt-losing diuretics, tobacco, non-steroidal anti-inflammatory drugs, and acetaminophen [16,17]. A thorough clinical evaluation and understanding of fluid and electrolyte balance are necessary to decide whether to initially correct hyponatremia with normal saline or much more slowly with hypertonic 3% saline for short periods.

In severe hyponatremia (sodium <120 mEq/L) with neurologic symptoms, a hypertonic solution of 3% saline should be administered. If neurologic symptoms are absent, the primary focus should be on a slow rate of sodium correction with intravenous fluids to achieve a rate that does not exceed 8-12 mEq/L per 24 hours. During the infusion of fluids, it is essential to monitor serum sodium levels every four to six hours. Sodium levels can be monitored hourly if there are severe derangements in sodium. The overarching objective is to correct serum sodium at a pace that does not exceed 0.5 mEq/hour [9,12]. The serum sodium level must be checked throughout the stabilization phase. Because vasopressin is crucial for maintaining the proper balance of salt and water, conivaptan, a vasopressin V1A/V2 receptor antagonist, may help treat acute euvolemic hyponatremia [3,15]. After CPM, new therapies for patients might be forthcoming. It has been observed that plasmapheresis and intravenous immunoglobulin injection improve results. Steroids, symptomatic medication for particular neurologic or neuropsychiatric symptoms, and psychotherapies or behavioral interventions, if psychogenic polydipsia is present, are additional potential treatments.

## Conclusions

CPM with carbamazepine-induced SIADH is extremely uncommon, with high morbidity and mortality. CPM remains a condition for neuropsychiatrists to be aware of, particularly given the multiple neuropsychiatric medication use, especially carbamazepine, and the psychiatric illnesses in which hyponatremia is a potential risk and requires close monitoring of serum electrolytes. Prevention is the key as current treatment rarely results in full recovery.
